# The PSMA-11-derived hybrid molecule PSMA-914 specifically identifies prostate cancer by preoperative PET/CT and intraoperative fluorescence imaging

**DOI:** 10.1007/s00259-020-05184-0

**Published:** 2021-01-23

**Authors:** Ann-Christin Eder, Mohamed A. Omrane, Sven Stadlbauer, Mareike Roscher, Wael Y. Khoder, Christian Gratzke, Klaus Kopka, Matthias Eder, Philipp T. Meyer, Cordula A. Jilg, Juri Ruf

**Affiliations:** 1 Department of Nuclear Medicine, University Medical Center Freiburg, Faculty of Medicine, University of Freiburg, Freiburg, Germany; 2 Division of Radiopharmaceutical Development, German Cancer Consortium (DKTK), partner site Freiburg, Freiburg, Germany and German Cancer Research Center, Heidelberg, Germany; 3 Division of Radiopharmaceutical Chemistry, German Cancer Research Center (DKFZ), Heidelberg, Germany; 4 Helmholtz-Zentrum Dresden-Rossendorf (HZDR), Institute of Radiopharmaceutical Cancer Research, Dresden, Germany; 5 Division of Radiooncology/Radiobiology, German Cancer Research Center (DKFZ), Heidelberg, Germany; 6 Department of Urology, University Medical Center Freiburg, Faculty of Medicine, University of Freiburg, Freiburg, Germany; 7 German Cancer Consortium (DKTK), partner site Dresden, Dresden, Germany; 8 Dresden University of Technology, Faculty of Chemistry and Food Chemistry, Dresden, Germany; 9 German Cancer Consortium (DKTK), partner site Freiburg, Freiburg, Germany

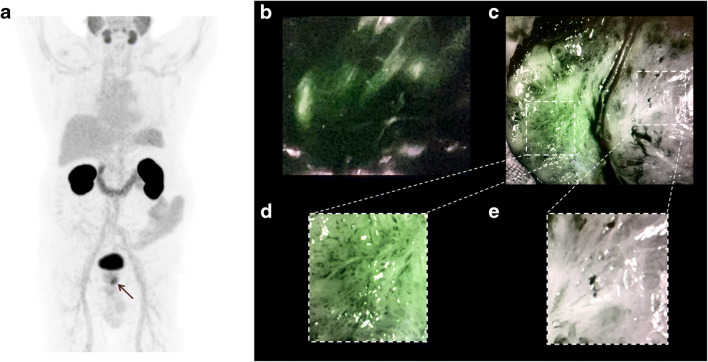



Resection of tumor tissue represents one of the standard curative treatment options for the clinical management of prostate cancer [1]. However, intraoperative localization and precise delineation of malignant tissue from surrounding healthy structures still remain challenging [2]. The development of PSMA-targeting hybrid molecules enabling the pre- and intraoperative detection of tumor tissue supported by both radioactivity (e.g., using DROP-IN technology) and fluorescence might help to overcome these limitations [3–5].

Here, we report for the first time preoperative PET/CT imaging and subsequent fluorescence-guided surgery aided by a PSMA-11-derived peptidomimetic PSMA-targeting hybrid molecule [6, 7].

A 71-year-old patient with high-risk prostate carcinoma (Gleason score 9 (4 + 5), initial PSA level 7 ng/ml) underwent preoperative PET/CT imaging with ^68^Ga-Glu-urea-Lys-(HE)_3_-HBED-CC-IRDye800CW (^68^Ga-PSMA-914) 1 h after intravenous tracer injection, revealing strong tracer uptake of the primary tumor located in the left prostate lobe (A, arrow).

DaVinci-assisted radical prostatectomy was subsequently performed the day after PET imaging under fluorescence guidance (PSMA-914 administration: intravenously 1 h prior to surgery). The primary tumor was clearly visualized by the specific fluorescence signal (green) and delineated from surrounding tissue (B). Consecutive ex situ fluorescence detection after radical prostatectomy (C) verified the intraoperative findings of tumor-specific hybrid molecule enrichment (D) resulting in high contrast to surrounding healthy structures (E).

Our initial experience with the novel PSMA-11-derived hybrid molecule PSMA-914 demonstrates its potential to precisely detect PSMA-expressing lesions pre- and intraoperatively.
